# Autonomous navigation and collision prediction of port channel based on computer vision and lidar

**DOI:** 10.1038/s41598-024-60327-9

**Published:** 2024-05-17

**Authors:** Zhan Zhang, NanWu Yang, YiJian Yang

**Affiliations:** 1 Southwest Research Institute for Hydraulic and Water Transport Engineering, Chongqing Jiaotong University, Chongqing, 400016 China; 2 Guangxi Beigang Planning and Design Institute Co., Ltd, Nanning, 530200 China

**Keywords:** Port channel, Autonomous navigation, Collision prediction, Computer vision, Laser radar, Materials science, Mathematics and computing

## Abstract

This study aims to enhance the safety and efficiency of port navigation by reducing ship collision accidents, minimizing environmental risks, and optimizing waterways to increase port throughput. Initially, a three-dimensional map of the port’s waterway, including data on water depth, rocks, and obstacles, is generated through laser radar scanning. Visual perception technology is adopted to process and identify the data for environmental awareness. Single Shot MultiBox Detector (SSD) is utilized to position ships and obstacles, while point cloud data create a comprehensive three-dimensional map. In order to improve the optimal navigation approach of the Rapidly-Exploring Random Tree (RRT), an artificial potential field method is employed. Additionally, the collision prediction model utilizes K-Means clustering to enhance the Faster R-CNN algorithm for predicting the paths of other ships and obstacles. The results indicate that the RRT enhanced by the artificial potential field method reduces the average path length (from 500 to 430 m), average time consumption (from 30 to 22 s), and maximum collision risk (from 15 to 8%). Moreover, the accuracy, recall rate, and F1 score of the K-Means + Faster R-CNN collision prediction model reach 92%, 88%, and 90%, respectively, outperforming other models. Overall, these findings underscore the substantial advantages of the proposed enhanced algorithm in autonomous navigation and collision prediction in port waterways.

## Introduction

Port channel is the key hub of global trade, so it is very important to ensure the safe navigation and collision prediction of ships. Port navigation is highly complex, influenced by maritime traffic density and meteorological conditions, and there are various obstacles^1^. The traditional navigation system relies on radar, Global Positioning System (GPS) and other technologies, but there are limitations in the complex port environment, such as difficulty in realizing high-precision perception and accurate collision prediction^2,3^. In addition, at present, most of the methods for detecting obstacles in port waterways rely on machine vision or radar images, but the shortcomings of using machine vision or radar to detect obstacles alone are very obvious^4^. Compared with radar, machine vision has many advantages, such as a large detection range, rich original information, accurate angle measurement, and strong target classification ability. However, using machine vision sensors alone leads to poor detection results in extreme weather such as fog, which seriously interferes with the effective acquisition of visual images^5,6^. When radar is used alone for target detection, although the speed of the target can be measured at the same time, it leads to low angular resolution, large signal loss and poor target classification ability. Therefore, no matter which target detection method is chosen, it is not the best choice, which greatly limits the ability of port channel to detect and identify obstacles. And it cannot meet the current needs of port channel obstacle identification^7,8^.

Firstly, regarding the application of computer vision and laser radar in autonomous navigation and collision prediction in port waterways, compared to traditional technologies like radar and GPS, computer vision and laser radar can offer more detailed and high-resolution environmental perception. Laser radar provides precise distance measurements, while computer vision can perform real-time analysis of visual information around ships, allowing for a more comprehensive understanding of the relationship between ships and their environment. Moreover, in handling complex scenes and multimodal information, computer vision demonstrates more robust capabilities than sensor technologies. Although laser radar is highly accurate in distance measurement, it may have limitations in certain situations, such as dense fog or intense light exposure. In contrast, computer vision systems, leveraging techniques like deep learning, can learn and adapt to various complex environments, enhancing perceptual performance under adverse weather conditions. In addition, Iran's “Morvarid” project uses positioning radar and detection equipment to research autonomous robots at sea. This also illustrates how radar technology assist in locating collisions within port navigation. Previous research has indicated that radar and laser monitoring technologies may face limitations in resolution and accuracy in certain scenarios. Particularly in complex environments, accurately identifying small-sized or densely packed targets may be challenging, impacting the performance of target detection and tracking. Radar systems may also experience significant performance degradation in adverse weather conditions like rain, snow, or fog due to electromagnetic wave interference. Laser monitoring systems may face limitations in atmospheric scattering and absorption. Moreover, radar and laser monitoring primarily rely on target reflection or scattering of electromagnetic waves, limiting their detection capabilities for non-cooperative targets such as potential threats or stealthy objects. In comparison to past research primarily based on a single sensor, this study achieves a more comprehensive perception of the port environment by integrating radar, laser, and image sensors, and enhancing the global awareness of the navigation system. In contrast to traditional RRT algorithms, this study introduces the artificial potential field method for optimization, generating safer and more effective navigation paths in dynamic waters. The incorporation of real-time decision-making algorithms enables the system to autonomously adjust navigation strategies based on real-time collision predictions. This is relatively uncommon in previous research and provides more flexible navigation strategies for the practical application of autonomous navigation systems.

The motivation of this study is to enhance the safety of port navigation by reducing collision accidents, lowering environmental risks, optimizing waterways, and ultimately increasing port throughput. The study focuses on three main objectives: firstly, an advanced path planning algorithm is employed to considerthe dynamic characteristics of vessels and environmental conditions to determine the optimal navigation path. Secondly, a collision prediction model using historical data and real-time sensor information is established to forecast potential collision risks. Lastly, a real-time decision-making algorithm is designed to allow the system to autonomously adjust navigation strategies based on collision prediction results and avoid potential collisions. In order to achieve these goals, visual perception technology is applied to process lidar data and camera images for a better understanding of the port channel environment. Specific applications include point cloud data processing, target detection, and tracking. Considering vessel dynamics, channel complexity, and safety requirements, the artificial potential field method is introduced to enhance the capability of the Rapidly-Exploring Random Tree (RRT) in generating optimal navigation paths. In collision prediction, K-Means clustering improves the Fast Region-based Convolutional Network (Fast R-CNN) algorithm, constructing a robust collision prediction model. This model uses historical data and real-time sensor information for training to predict future trajectories of other vessels and obstacles.

The study utilizes a comprehensive fusion of radar, lidar, and image sensor data. Lidar scanning is employed to obtain a three-dimensional map of the port channel, followed by environmental perception through visual perception technology. This sensor data fusion enhances a comprehensive understanding of the port environment, facilitating more accurate navigation and collision prediction. The introduction of a real-time decision-making algorithm allows the system to autonomously adjust navigation strategies based on collision prediction results, promptly avoiding potential collision events. However, there are technical challenges, such as effectively integrating data from radar, lidar, and image sensors. It needs to overcome heterogeneity, noise issues, and real-time requirements of different sensors. High-precision collision prediction is crucial for avoiding potential collisions, particularly in complex water environments where accurately predicting the future trajectories of other vessels and obstacles is challenging. The study combines advanced image processing and autonomous navigation algorithms to improve the safety and efficiency of port navigation, reducing the risk of collision accidents and safeguarding lives and the environment. The innovation lies in the integration of multisensory data, enhanced path planning, and efficient collision prediction, providing a comprehensive and viable solution for autonomous navigation and collision prediction in port channels. The expected outcome is a substantial improvement in the safety and efficiency of port navigation.

## Literature review

The discipline of ship navigation is becoming more and more interested in autonomous navigation technologies. A new autopilot system that connected wave height prediction and ship driving was put forth by Lou et al. in^9^. The wave height may be precisely anticipated using Long Short-Term Memory (LSTM), and the ship can change its path in real-time to always travel in the region with the lowest wave height^9^. Gucma^10^ used the technique of computer-simulating ship traffic flow and split the approach channel into one-way and two-way segments in the best possible way. A unique two-stage simulation optimization method was used to find the ideal port entrance, steering pool, and port pool characteristics, and simulation tests were run on a three-dimensional visual ship manoeuvring simulator. The study has established the outer container terminal in Swinnuisi’s best specifications, and the terminal’s anticipated annual container handling capacity was 1.5 million TEUs. An ocean-going container ship^10^ with a length of 400 m and a width of 60 m was anticipated to be operated by the port. In their investigation of a reliable control system for unmanned surface vehicles in urban waterways, Cortes-Vega et al. suggested using a visual odometer to assess the position of the vehicles rather than conventional sensors^11^. Mansuy et al. simulated the turning manoeuvres of two typical inland river shipping vessels under different hydrometeorological conditions in real time, and proposed a step-by-step method to select the optimized turning pool geometry according to the field conditions. This step-by-step design of the turning pool method can reduce the real-time simulation required for upgrading the waterway network^12^. Sinohara et al. analyzed the specific needs of autonomous ships for external and environmental information in restricted pilotage waters, and put forward targeted technical solutions in Paranagua and Antonina, Brazil^13^. Nzengu et al. analyzed the regulatory framework related to the operation and testing of unmanned Inland Waterway (IWW) ships in Flanders. A three-stage strategy is put forward as a road map for formulating the regulatory framework to adapt to IWW autonomous ships more widely^14^.

Computer vision technology and lidar have made remarkable progress in the field of navigation. Hu et al. used lidar to monitor the visibility of sea fog in Beilun area of Zhoushan Port in Ningbo, China, and compared the data of lidar with that of forward scattering visibility sensor. The results showed that the visibility lidar instrument had advantages in sea fog monitoring, and the correlation between lidar instrument data and forward scattering sensor data proved the practicability and potential of lidar in sea fog detection^15^. Lu et al. used Automatic Identification System (AIS), video surveillance, laser radar and other intelligent sensing technologies to realize automatic and accurate collection of channel traffic data, and built an integrated platform of existing ship channel traffic monitoring system in Yancheng, which integrated multi-dimensional sensing, fusion processing and statistical analysis^16^. Tak et al. monitored the beach width and beach profile along the east coast of Korea by unmanned aerial vehicles and ground-based lidar. The results showed that the plane layout of port facilities concentrated waves and increased the number of sediments northward^17^. Hake et al. used multi-sensor system to scan the above-water and underwater port structures, and used Visual Geometry Group19 (VGG19) deep neural network and local abnormal factors to identify the grid network of point clouds on the steel sheet pile wall. The results showed that the accuracy of VGG19 deep neural network was 8.95%^18^. Marchel et al. used extended Kalman filter and two-dimensional range bearing to evaluate the positioning accuracy of ships following a constant course and speed in the port approach channel, which showed that the adopted algorithm could be successfully used to plan their deployment to ensure the minimum accuracy requirements of navigation marking service in positioning navigation marks on the port approach channel and under restricted conditions^19^.

Channel collision is a serious safety problem, so it is very important to study collision prediction and prevention methods. Upadhyay et al. used the collaborative method of computer vision to track the target according to the specific position of interest in the image. Compared with the actual measurement, the test results on the framework of quadrotor UAV achieved 99% positioning accuracy^20^. Padmaja et al. proposed a collision warning system for self-driving cars based on a new point-to-pixel multi-sensor data fusion algorithm, and used MobileNet SSD to classify targets. The results showed that the root mean square error and mean absolute error of the proposed fusion algorithm were 2.93 mm and 802.83 mm lower than those of the stereo camera and the two-dimensional lidar sensor respectively^21^. Miao et al. proposed a UAV obstacle identification based on airborne lidar and an improved density-based noisy application spatial clustering algorithm, and the experiments proved the effectiveness of the proposed algorithm in identifying the invading mobile state^22^. Guan et al. introduced a new multi-model full traffic trajectory data, and measured the fluctuation of pedestrian speed by computer lidar and computer vision respectively. Compared with the data based on computer vision, the current trajectory data based on lidar showed a wider detection range and was less affected by poor lighting conditions^23^.

The above literature shows that autonomous navigation technology, computer vision and lidar technology are widely used in the field of navigation, which provides strong support for improving the safety and efficiency of ship navigation. These studies cover wave height prediction, channel optimization, unmanned surface vehicle control, collision prediction and prevention, environmental perception and obstacle detection. However, there are still some challenges to be solved, such as environmental changes and sensor errors, to further improve the performance of the navigation system. This study continues to explore innovative methods to promote the development of navigation field and improve the robustness and applicability of navigation system.

## Research methodology

### Visual perception technology and port channel environment perception

**Figure 1:**
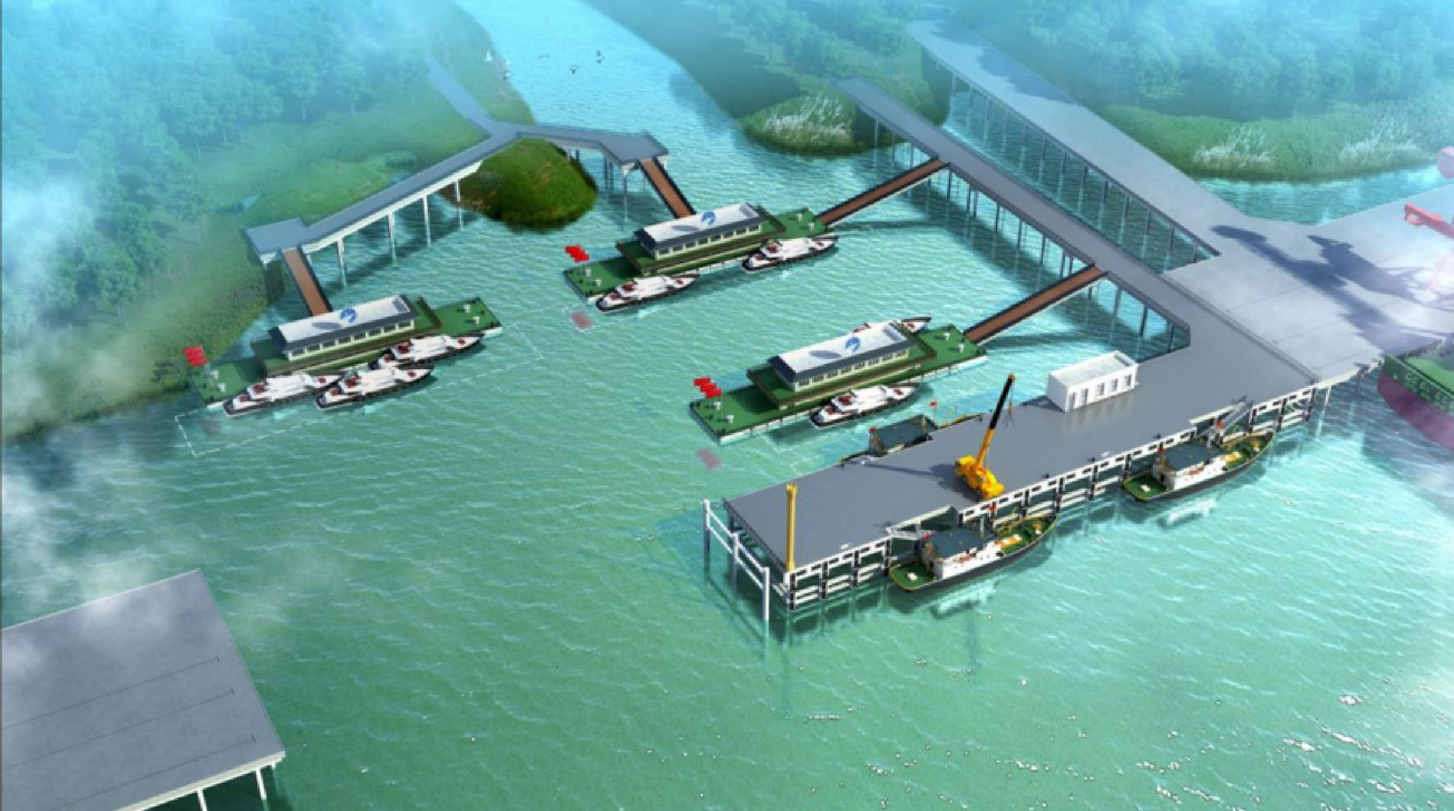
Three-dimensional map of port channel.

In port channel perception, point cloud data are obtained from lidar sensors. These data include a large number of discrete three-dimensional points, and each point represents a position in space. Point cloud data need to be processed and analyzed to create a three-dimensional map of the port channel. The steps of processing point cloud data include data acquisition and preprocessing, point cloud segmentation and grouping, and 3D map generation. The original point cloud data is obtained from the lidar sensor, and then the preprocessing steps such as denoising, filtering and coordinate transformation are carried out to prepare the data for subsequent processing. According to the characteristics of point cloud, point cloud data is divided into different objects or features, such as water surface, other ships, shore and obstacles. The processed point cloud data are combined to generate a three-dimensional map of the port channel, including the location information of waterways, port facilities and other targets^24,25^. Figure 1 shows the three-dimensional map of the port channel.

**Figure 2:**
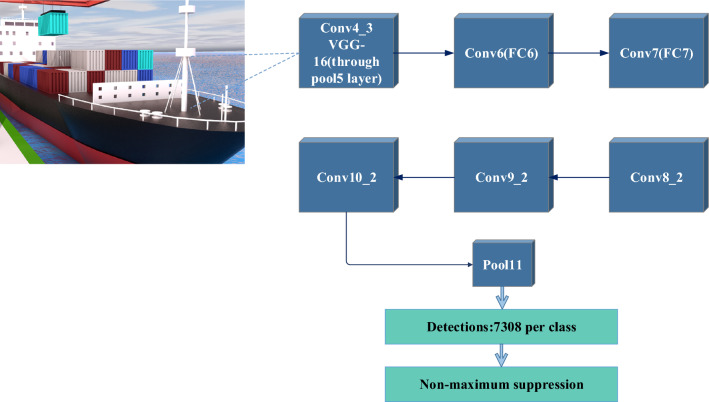
SSD structure.

Finding the whereabouts of other ships and obstructions in the port channel is crucial. Target recognition and tracking technology is used in this study to keep an eye on nearby ships and objects in real time. The Single Shot MultiBox Detector (SSD) technique is used in this procedure. SSD is a convolutional neural network-based target detection technique. To detect objects of various sizes and forms, it employs multi-scale feature maps, and to detect targets of various shapes, it employs multiple anchor frames. By performing a convolution operation on feature maps with various levels, SSD achieves multi-scale target detection^26^. The SSD structure is shown in Fig. 2.

In Fig. 2, SSD is based on Visual Geometry Group 16 (VGG16) network, and the fully connected layer is modified as convolution layer, and then four convolution layers are added, namely, conv6, conv7, conv8_2, conv9_2, conv10_2, conv11_2, conv4_3, conv7, conv8_2 and conv. Conv10_2 and conv11_2 are used as the detection heads of the network, and the detection results are obtained through the final Non-Maximum Suppression (NMS) non-maximum suppression. SSD starts from Conv4_3 and extracts feature maps. The number of prior frames set on each feature map is different, and the setting of prior frames follows the rule of linear increase, that is, the size of feature map decreases and the scale of prior frames increases linearly. Equation (1) shows the setting of prior frames:

1 $${s}_{k}={s}_{min}+\frac{{S}_{max}-{s}_{min}}{m-1}\left(k-1\right),k\in [1,m]$$

In Eq. (1), $m$ is the number of feature graphs, ${s}_{k}$ is the ratio of the prior frame size to the picture, and ${s}_{min}$ and ${S}_{max}$ are the minimum and maximum values of the ratio respectively. The center point of the prior box of each pixel is distributed in the center of each pixel, and the calculation is shown in Eq. (2):

2 $$\left(\frac{i+0.5}{\left|{f}_{k}\right|},\frac{j+0.5}{\left|{f}_{k}\right|}\right),i,j\in [0,\left|{f}_{k}\right|]$$

In Eq. (2), $\left|{f}_{k}\right|$ represents the size of the feature map. In the process of prediction, the model is predicted by using bounding_box. The purpose of bounding_box regression is: given $\left({P}_{x},{P}_{y},{P}_{w},{P}_{h}\right)$, find the mapping $f$, so that $f\left( {P_{x} ,P_{y} ,P_{w} ,P_{h} } \right) = \left( {G_{x}^{ \wedge } ,G_{y}^{ \wedge } ,G_{w}^{ \wedge } ,G_{h}^{ \wedge } } \right)$ can be obtained. $G$ is ground truth and $G^{ \wedge }$ is bounding box. Eqs. (3–6) show the border regression process:

3 $$ G_{x}^{ \wedge } = P_{w} \frac{{\left( {G_{x}^{ \wedge } - p_{x} } \right)}}{{P_{w} }} + P_{x} ,\left( 1 \right) $$

4 $$ G_{y}^{ \wedge } = P_{h} \frac{{\left( {G_{h}^{ \wedge } - p_{y} } \right)}}{{p_{h} }} + P_{y} ,\left( 2 \right) $$

5 $$ G_{w}^{ \wedge } = P_{w} {\text{exp}}\left( {log\frac{{\left( {G_{w}^{ \wedge } } \right)}}{{P_{w} }}} \right),\left( 3 \right) $$

6 $$ G_{h}^{ \wedge } = P_{h} {\text{exp}}\left( {log\frac{{\left( {G_{h}^{ \wedge } } \right)}}{{p_{h} }}} \right),\left( 4 \right) $$

In the above equation, $\left( {G_{x}^{ \wedge } ,G_{y}^{ \wedge } ,G_{w}^{ \wedge } ,G_{h}^{ \wedge } } \right)$ is the position offset and scale transformation of Bounding_box relative to prior_box. In an ideal state, the position offset and scale transformation of bounding_box relative to prior_box is shown in Eqs. (7–10):

7 $${t}_{x}=\frac{({G}_{x}-{P}_{x})}{{P}_{w},(6)}$$

8 $${t}_{y}=\frac{({G}_{y}-{P}_{y})}{{P}_{h},(7)}$$

9 $${t}_{w}={\text{log}}\frac{\left({G}_{w}\right)}{{P}_{w}},(8)$$

10 $${t}_{h}={\text{log}}\frac{\left({G}_{h}\right)}{{P}_{h}},(9)$$

Through the above equation, the Loss function can be obtained, through reducing the loss, the convergent position offset and scale transformation can be finally obtained, and the final predicted bounding_box can be obtained through decoding with prior_box. Equation (11) shows the loss function:

11 $${\text{Loss}}({\text{x}},{\text{c}},{\text{l}},{\text{g}})=\frac{1}{N}({L}_{conf}\left(x,c\right)+\alpha {L}_{loc}\left(x,l,g\right))$$

In Eq. (11), $N$ is the number of positive samples in the prior frame, $c$ is the predicted value of category confidence, ${\text{l}}$ is the predicted value of the position corresponding to bounding_box in the prior frame, $g$ is the position parameter of ground truth, and $\alpha$ is taken as 1 through cross-verification. The position function of ${L}_{loc}\left(x,l,g\right)$ is shown in Eqs. (12, 13):

12 $${L}_{loc}\left(x,l,g\right)=\sum_{i\in pos}^{N}\sum_{m\in \left\{cx,cy,w,h\right\}}{x}_{ij}^{p}{Smooth}_{L1}\left({l}_{i}^{m}-{\widehat{g}}_{j}^{m}\right)$$

13 $${Smooth}_{L1}\left(x\right)=\left\{\begin{array}{ll}0.5{x}^{2}, & \quad if\;\left|x\right|<1\\ \left|x\right|-0.5, & \quad otherwise\end{array}\right.$$

In the above equation, ${l}_{i}^{m}$ and ${\widehat{g}}_{j}^{m}$ are the position parameters after encode, ${x}_{ij}^{p}$ is the $i$ th prior_box matching with the $j$ th gt_box, and the category of gt_box is $p$, with a value of 1, otherwise with a value of 0. The position loss function is only for positive samples. For every prior_box matching gt_box, the difference between the offset and scaling scale of bounding_box and that of gt_box is calculated by using ${Smooth}_{L1} \left(x\right)$ loss, and the optimization is achieved by reducing its value. L2 regularization in Conv4_3 is used, as shown in Eq. (14):

14 $${y}_{i}={L}_{2}Norm\left(x\right)=\frac{{x}_{i}}{\sqrt{\sum_{k=1}^{n}{x}_{k}^{2}}}$$

The Conv4_3 layer has a different feature scale compared with other layers. L2 regularization technology is used to normalize the feature of each pixel in conv4_3 feature map to 20 to ensure that there is little difference with the following layers. In the process of identifying the positions of other ships and obstacles, SSD algorithm inputs the preprocessed point cloud data and camera images into SSD algorithm. SSD algorithm extracts features from input data through CNN for target detection. The feature map is used with the anchor frame to locate the target and determine the locations of other ships and obstacles. The target tracking algorithm updates the target's position information in real-time for path planning and collision prediction^27^.

### Path planning algorithm and optimal navigation path generation

In the aspect of path planning, considering the dynamic characteristics of the ship, the complexity of the channel environment and the safety requirements, the improved Rapidly-Exploiting Random Trees (RRT) is adopted to generate the best navigation path^28,29^. Table 1 shows the principle of RRT algorithm.

**Table 1:** Principle of RRT algorithm.

GENERATE_RRT$$(x_{init} ,K,\Delta \,t)$$
1. T.init($${x}_{init})$$;
2. For k=1 to K do
3. If ($$\Vert {x}_{new}-{x}_{goal}\Vert <d)$$
4. Break;
$$5.\,{x}_{rand}\leftarrow RANDOM\_STATE();$$
$$6.\,{x}_{near}\leftarrow NEAREST\_NEIGHBOR({x}_{rand},T);$$
$$7.\,u\leftarrow SELECT\_INPUT({x}_{rand},{x}_{near});$$
$$8. $$$$x_{new} \leftarrow NEW\_STATE\left( {x_{near} ,u,\Delta \,t} \right)$$
9. Judge $$(x_{new} )$$$$;$$
10. If (judge ($${x}_{new})==false\,and\,colision\,free()==false)$$
11. Continue;
12. T.add_vertex ($${x}_{new});$$
13. T.add_edge ($${x}_{near}$$,$${x}_{new},u);$$
14. Return T

In Table 1, $RANDOM\_STATE()$ function generates random points within the set environment, $NEAREST\_NEIGHBOR()$ function traverses the random tree to find the node closest to the random point. $SELECT\_INPUT()$ function expands the random tree according to the set value, $NEW\_STATE()$ function generates ${x}_{new}$, judge (${x}_{new}$) function determines whether the newly generated node satisfies non holonomic constraints, T.add_Vertex () insert ${x}_{new}$, T.add_Edge () adds an edge between ${x}_{near}$ and ${x}_{new}$, do not add new nodes in this loop. Regenerate x in the next loop_New, and then make a judgment if it belongs to ${X}_{free}$, then keep the new node. In summary, after adding ${x}_{new}$, when adding a new node, it needs to be judged twice, namely obstacle detection and non-holonomic constraint detection. Only when both meet the requirements can a new node be added^30^.

**Figure 3:**
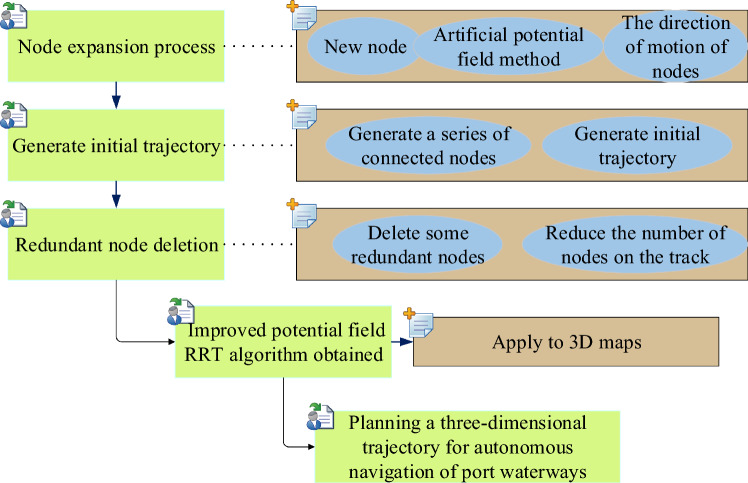
Autonomous navigation process of port channel based on improved RRT algorithm.

However, the initial path generated by RRT algorithm is completely random, and it may not be possible to search for navigation tracks in maps with many obstacles. In this paper, the concept of artificial potential field method is combined, and the generated track is more in line with the requirements of safety and smoothness through the action of repulsion and gravity. By deleting redundant nodes, the efficiency and feasibility of the track are further improved. Figure 3 shows the autonomous navigation process of port channel based on improved RRT algorithm.

Figure 3 shows an updated RRT method that starts from a beginning point, creates additional nodes in a random manner, and tries to connect the new nodes to the preexisting tree structure. The artificial potential field approach is used to expand nodes, and each node is subjected to the attraction of the target point and the repulsion of barriers, ensuring that the node proceeds to the target point along the safest path. Through the process of node expansion, the algorithm gradually generates a series of connected nodes, forming the initial track. In order to make the track smoother and safer, some redundant nodes are deleted by line-of-sight algorithm. The line-of-sight algorithm checks the nodes on the track. If there is no obstacle between two nodes, the intermediate node between them can be deleted. Through the above steps, the improved RRT algorithm of potential field is obtained. This algorithm generates a smooth, safe flight path with fewer nodes, which can be used for navigation and path planning. Finally, the improved potential field RRT algorithm is applied to the three-dimensional map to plan the three-dimensional track of the autonomous navigation process of the port channel.

### Collision prediction model and training process

**Figure 4:**
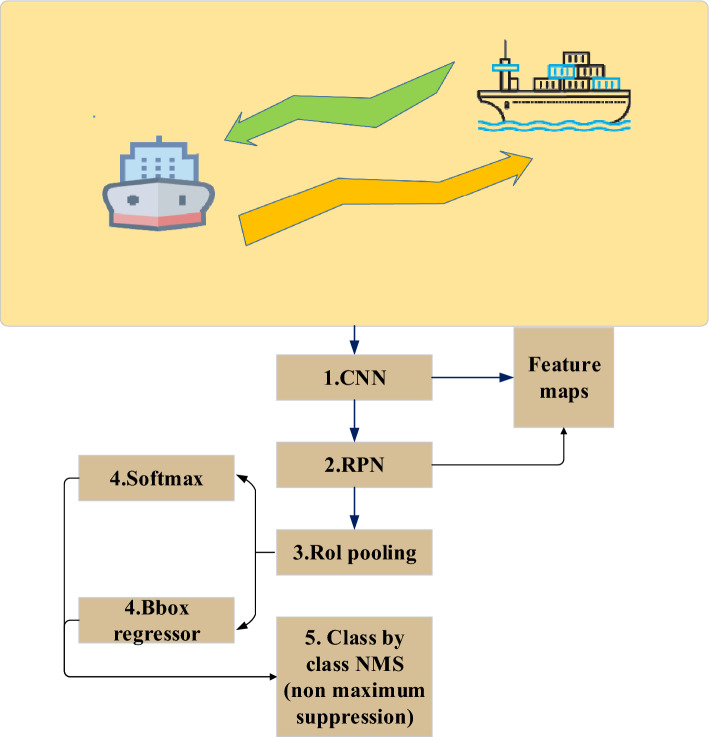
Structure of Faster R-CNN.

In the autonomous navigation system of port channel, the construction and training process of collision prediction model is very important to ensure that ships can safely avoid collision and plan the best path. In this study, a collision prediction model is created using an upgraded FAST Region-based Convolutional Neural Network (R-CNN). A popular deep learning model for target identification and object recognition is Faster R-CNN, which can identify objects in photos and pinpoint their locations. The Faster R-CNN's construction is depicted in Fig. 4.

In Fig. 4, Fast R-CNN consists of two main components: CNN and Region Proposal Network (RPN). CNN is used to extract features from input images. These feature maps contain different levels of information in the image and are used for subsequent target detection tasks. RPN is used to generate candidate regions. It slides the window on the convolution feature map of the backbone network and outputs the suggested target box through classification and regression header. Each suggestion box is accompanied by a candidate box score for subsequent screening. The RoI pooling layer is used to cut and standardize candidate frames of different sizes into feature maps of the same size for input into the subsequent classification and regression head network. These networks receive the characteristic map of RoI pool as input, and carry out target classification and position regression. The classification header is used to determine whether the candidate frame contains the target object, and the regression header is used to adjust the position of the candidate frame. In the training process of Faster R-CNN, the loss calculation of the network is shown in Eqs. (15, 16):

15 $$L(\left\{{p}_{i}\right\},\left\{{t}_{i}\right\})=\frac{1}{{N}_{cls}}\sum_{i}{L}_{cls}\left({p}_{i},{p}_{i}^{*}\right)+\lambda \frac{1}{{N}_{reg}}\sum_{i}{{p}_{i}^{*}L}_{reg}\left({t}_{i},{t}_{i}^{*}\right)$$

16 $${L}_{reg}\left({t}_{i},{t}_{i}^{*}\right)=\sum_{i\in \left\{x,y,w,h\right\}}{smooth}_{L1}({t}_{i}-{t}_{i}^{*})$$

**Figure 5:**
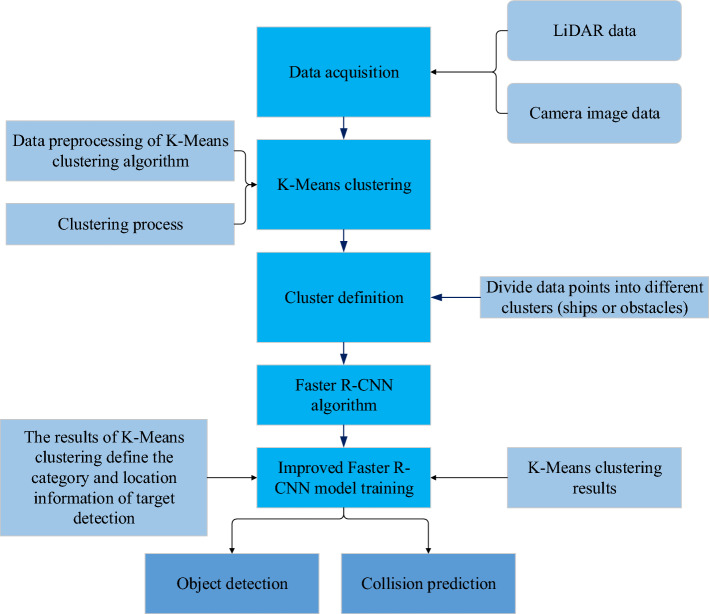
Collision prediction model based on improved Faster R-CNN algorithm.

In the above equation, $i$ is the anchors index. ${p}_{i}$ is the positive softmax probability. ${p}_{i}^{*}$ is the corresponding GT predict probability. $t$ is the predict bounding box, and ${t}_{i}^{*}$ is the corresponding GT box of the positive anchor. ${L}_{cls}$ is the softmax loss calculated by RPN_cls_loss layer, which is used to classify anchors as positive and negative network training, and ${L}_{reg}$ is the soomth L1 loss calculated by RPN_loss_bbox layer, which is used to train the bounding box regression network. In the actual port navigation collision prediction, Faster R-CNN usually needs large-scale tag data for training, but in a specific port channel environment, it may need more model adaptability to adapt to different meteorological conditions, port structures and ship types. In this study, K-Means clustering algorithm is used to improve Faster R-CNN algorithm for port channel collision prediction, and K-Means algorithm is used to cluster lidar data and image data, and the data points are divided into different clusters. These clusters can represent different types of ships and obstacles. In Faster R-CNN, the results of K-Means clustering are used to define the category and location information of target detection. This can improve the accuracy of target detection. Finally, the improved Faster R-CNN model is used for training, so that the model can detect targets and predict collisions according to the K-Means clustering results. Figure 5 shows the collision prediction model of the improved Faster R-CNN algorithm.

## Results and discussion

### The result of SSD algorithm to identify the position of ships and obstacles

**Figure 6:**
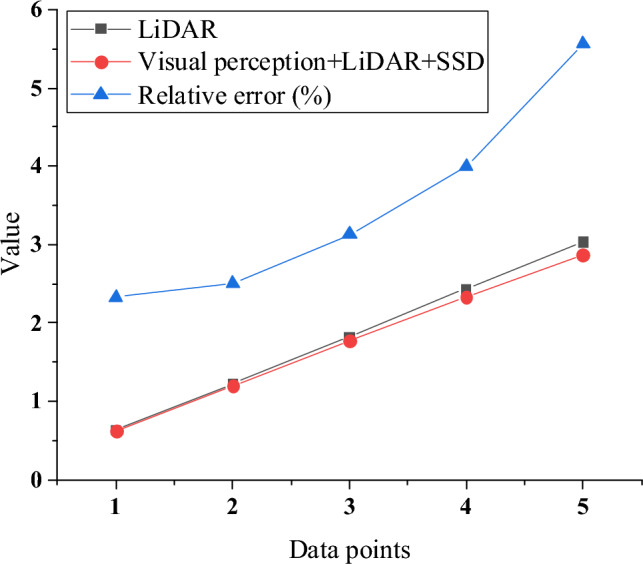
Comparison of SSD algorithm to identify the position of ships and obstacles with the actual results.

Figure 6 shows the comparison results between SSD algorithm and actual results in identifying the positions of ships and obstacles. In Fig. 6, in the case of testing data point 1 to data point 3, the performance of using visual perception + lidar + SSD algorithm is better than using lidar only. The relative errors are 2.33%, 2.5% and 3.13% respectively. This shows that SSD algorithm has achieved good results in identifying the positions of ships and obstacles, and its accuracy is relatively higher. With the increase of data points, the relative error increases gradually. In the case of data point 5, the relative error reaches 5.56%. This may be because the performance of visual perception + lidar + SSD algorithm is challenged in a longer distance or in a more complex environment, and the error increases slightly. Generally speaking, visual perception + lidar +SSD algorithm performs well in ship and obstacle location recognition, and its performance is better than that of using lidar only.

The experimental results indicate that the combination of visual perception, lidar, and SSD algorithm performs better in short distances compared to using only lidar. This suggests that the integrated use of multisensor data (visual and lidar) enhances the accuracy of ship and obstacle position identification. The effective fusion of information from multiple sources allows the model to more accurately capture target positions in short distances. Although there is a slight increase in relative errors in some cases, the overall performance remains superior to the scenario using only lidar. Future improvements could involve introducing more training data to enhance generalization performance or adjusting algorithm parameters to balance performance under different distances and environmental conditions.

### Improvement of navigation path generated by RRT by artificial potential field method

**Figure 7:**
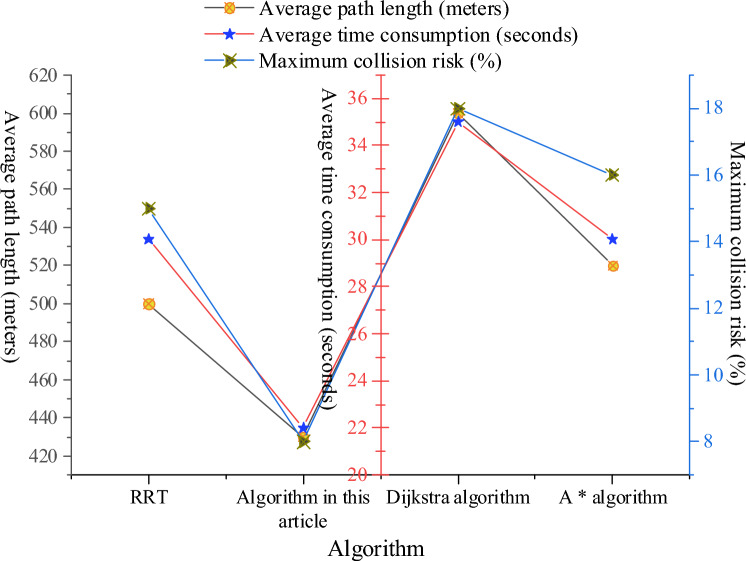
Improvement of navigation path generated by RRT by artificial potential field method.

The outcome of enhancing RRT to generate a navigation path using an artificial potential field approach is shown in Fig. 7. Figure 7 illustrates a considerable improvement in average path length and average time consumption between the RRT improved by the artificial potential field method and the conventional RRT algorithm. Average time consumption decreases from 30 to 22 s, and average journey length decreases from 500 to 430 m. Thus, the enhanced algorithm suggested in this study provides shorter and quicker travel paths and boosts navigational effectiveness. The RRT improved by artificial potential field method also shows obvious advantages in the maximum collision risk. The maximum collision risk is reduced from 15% of the traditional RRT algorithm to 8%. This shows that the algorithm in this study has made remarkable progress in reducing collision risk and improving navigation safety. Compared with Dijkstra algorithm and A* algorithm, RRT improved by artificial potential field method is competitive in average path length and average time consumption, which provides more efficient and safer navigation path planning for autonomous navigation of port channels.

Compared to Dijkstra’s algorithm and A* algorithm, the RRT algorithm improved through the artificial potential field method demonstrates competitiveness in both average path length and average time consumption. This implies that the enhanced RRT algorithm is more advantageous in terms of efficiency for navigation path planning compared to traditional algorithms. This provides a more efficient and secure planning path for autonomous navigation in port channels. The RRT algorithm improved through the artificial potential field method exhibits enhancements in various aspects, including path length, time consumption, and collision risk. This comprehensive improvement positions the enhanced algorithm as a viable choice in autonomous navigation systems, particularly in scenarios where rapid, secure, and low collision risk navigation paths are required.

### Performance results of improving the collision prediction model of faster R-CNN based on K-means clustering

**Figure 8:**
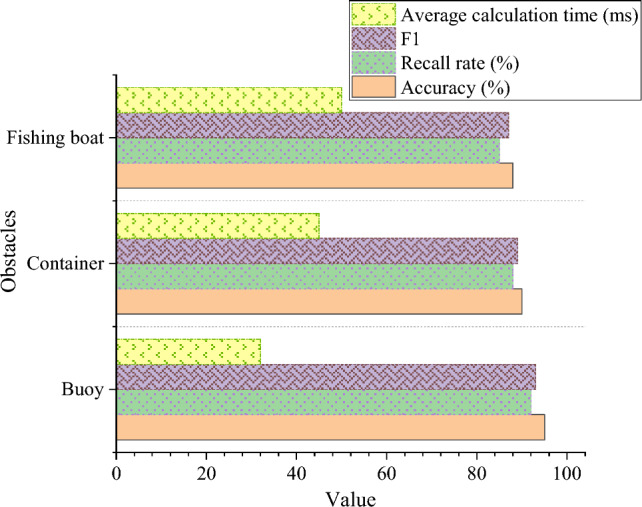
Correlation analysis results between digital platform and corporate social responsibility.

The study evaluates the model performance using accuracy, recall, and F1 score. Higher values for accuracy and recall, closer to 1, indicate better precision or recall rates. The F1 score ranges from 0 to 1, with 1 being the maximum value and 0 the minimum. A higher precision and recall are desirable. In the range of 0 to 1, a higher F1 score is preferable. Figure 8 shows the performance results of the collision prediction model improved by Faster R-CNN based on K-Means clustering. It shows that for the “buoy” type obstacle, the accuracy of the model is 95%, the recall rate is 92%, the F1 score is 93%, and the average calculation time is 33 ms. This shows that the model has high performance in detecting and locating buoy obstacles, and has high precision and recall rate. For the “container” type of obstacles, the performance of the model is relatively good, while for the “fishing boat” type of obstacles, the performance of the model is relatively low, with an accuracy of 88%, a recall rate of 85%, a F1 score of 87% and an average calculation time of 50 ms. This shows that the performance of the model is relatively low in detecting and locating obstacles of fishing boats. Generally speaking, the improved Faster R-CNN collision prediction model based on K-Means clustering shows different performance levels under different types of obstacles. The model has excellent performance in detecting buoy obstacles.

**Figure 9:**
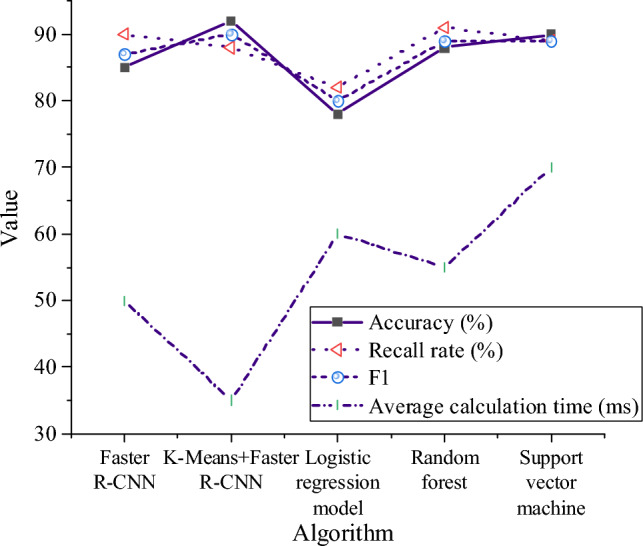
Performance comparison of different collision prediction models.

Figure 9 shows the performance comparison of different collision prediction models. In Fig. 9, the K-Means + Faster R-CNN algorithm proposed in this paper shows significant advantages in accuracy, recall and F1 score, and has higher performance compared with other collision prediction models. Its accuracy is 92%, the recall rate is 88%, and the F1 score is 90%, so it is one of the best models. Random forest model and support vector machine model also show high performance in accuracy, recall and F1 score, but they are slightly lower than K-Means + Faster R-CNN model. The performance of these models is better than that of Faster R-CNN and Logistic regression models. Logistic regression model is relatively low in accuracy, recall and F1 score, and its performance is the worst. Meanwhile, its average calculation time is also long, 60 ms. On the whole, K-Means + Faster R-CNN algorithm has obvious advantages over other collision prediction models in terms of performance index and calculation efficiency, and it is a better collision prediction model.

Taking a comprehensive view, the K-Means + Faster R-CNN algorithm demonstrates superiority in both performance and computational efficiency, making it a more outstanding collision prediction model. While other models perform well, they may not surpass K-Means + Faster R-CNN in certain performance indicators. The experimental results in Figs. 8, 9 indicate that the Faster R-CNN model improved by K-Means clustering exhibits excellent performance under different types of obstacles, particularly showing significant improvement in collision prediction.

## Conclusion

The successful integration of computer vision and Lidar technology enables effective perception and analysis of the port channel environment. The improved RRT algorithm, employing the artificial potential field method for path planning, significantly reduces average path length and time consumption, leading to a substantial improvement in navigation efficiency and safety. Additionally, the collision prediction model based on K-Means clustering and Faster R-CNN outperforms other models, demonstrating excellent adaptability to various obstacle scenarios. While the proposed algorithmic enhancements represent a significant breakthrough in the field of autonomous navigation and collision prediction in port channels, acknowledging the limitations of this study is crucial. Future studies should focus on refining path-planning algorithms to adapt to more complex environmental conditions and consider the dynamic characteristics of vessels. Furthermore, there is room for optimization in the collision prediction model to enhance its adaptability to a broader range of obstacles. Although this study marks significant progress, the identified constraints highlight areas for future exploration and improvement. Ongoing research can further refine the proposed algorithms to ensure their applicability to various dynamic maritime scenarios, ultimately advancing the field of autonomous navigation and collision prediction in port channels.

### Supplementary Information

Supplementary Tables.

## Data Availability

All data generated or analysed during this study are included in this published article [and its supplementary information files].
